# Assessment of Oral Health and Healthy Habits in Adult Patients with Congenital Hemophilia

**DOI:** 10.1055/s-0042-1743156

**Published:** 2022-05-02

**Authors:** Sylwia Czajkowska, Joanna Rupa-Matysek, Lidia Gil, Anna Surdacka

**Affiliations:** 1 Department of Conservative Dentistry and Endodontics, Poznan University of Medical Sciences, Poznan, Poland; 2 Department of Hematology and Bone Marrow Transplantation, Poznan University of Medical Sciences, Poznan, Poland

**Keywords:** Hemophilia A, Hemophilia B, Oral hygiene, Oral health, Dental caries

## Abstract

**Objectives** The objective of our study was to assess the current knowledge, the oral health status, and the pro-health needs of patients with hemophilia.

**Materials and Methods** The study included 77 patients with known hemophilia. The study included the assessment of dental indicators related to caries incidence (decayed, missing, and filled teeth [DMFT] and decayed, missing, and filled permanent teeth or surfaces [DMFS]), caries treatment (treatment index), oral hygiene status (Approximal Plaque Index [API] and simplified oral hygiene index [OHI-S]), and periodontal status (bleeding on probing [BoP]). A questionnaire addressed symptoms of hemorrhagic diathesis and health-promoting habits. The influence of routine management (“on-demand” vs. prophylactic therapy), type of hemophilia (A, B), and severity of clotting factor deficiency (mild, moderate, or severe) on oral health was assessed.

**Statistical Analysis** The tests used in the study were Shapiro–Wilk, Mann–Whitney, Kruskal–Wallis, and Dunn's tests. The level of significance was set at *p* < 0.05.

**Results** Patients with hemophilia showed a higher prevalence of caries compared with patients without hemorrhagic diathesis (DMFT 14 and 9, and DMFS 30 and 15, respectively), and they also presented a higher bleeding index value during probing and worse effectiveness of hygienic interdental procedures. The type of therapy and secondary prophylaxis used and the type and severity of hemophilia did not affect the oral health status.

**Conclusions** Patients with hemophilia have an increased risk of developing dental caries. Undertaking educational activities related to the prevention of oral diseases is necessary to improve oral health.

## Introduction

Congenital hemophilia is a bleeding disorder caused by a deficiency in one of the blood clotting factors. According to data published by the Ministry of Health in the National Program for the Treatment of Patients with Hemophilia and Related Hemorrhagic Diatheses for 2019 to 2023, it is estimated that hemophilia is the most common hemorrhagic diathesis in Poland. 1 The condition occurs with a frequency of 1 in 5,000 male births for hemophilia A (HA) and 1 in 30,000 male births for hemophilia B (HB) 1 and is inherited in an X-linked recessive disorder. This means that men, and possibly homozygous women, are affected. In HA and HB, the level of deficient clotting factor VIII (FVIII) and IX (FIX), 2 respectively, correlates with the severity of symptoms, and distinction is, therefore, made between severe (less than 1% factor activity, which corresponds to <0.01 IU/mL), moderate (1–5% factor activity), and mild hemophilia (more than 5% factor activity). The clinical presentation of HA and HB is similar and can be potentially life-threatening. The disease is characterized by excessive, prolonged, or spontaneous bleeding or bleeding after invasive procedures, even after minor surgical procedures such as dental extractions, even in patients with mild forms of the disease. Bleeds occur, among others, in the joints and muscles (especially in the severe form of hemophilia), the central nervous system, the genitourinary system, or the digestive system. 3 4 Patients with hemophilia report the presence of hematomas, as well as prolonged bleeding after minor cuts or injuries. Bleeding may also occur in the oral cavity—for example, spontaneous bleeding from the gums or excessive or prolonged bleeding after dental procedures. Repeated, spontaneous joint hemorrhages cause joint destruction and the development of hemophilic arthropathy. The occurrence of pseudo-tumors in the course of hemophilia has also been reported in the literature 5 6 7 8 —most often in the long bones or pelvis and less often in the craniofacial region. 6 8 Moreover, attempts were made to assess the influence of congenital coagulation disorders on the development of caries and other oral diseases; however, no unequivocal research results were obtained. One consequence of untreated caries is inflammation of the pulp and apical tissues, leading to tooth loss. Due to the higher risk of bleeding complications during dental procedures, assessment of the health of the oral cavity and determination of dental health needs play a particularly important role in this group of patients.

Laboratory tests in patients with hemophilia showed a decrease in the activity of one of the clotting factors (FVIII or FIX) and a prolonged activated partial thromboplast in time with correction in mixing studies. Prothrombin time, bleeding time, and platelet count remain normal. The optimal treatment is an intravenous infusion of a deficient clotting factor concentrate to reduce bleeding and minimize the risk of complications. Earlier therapies were associated with a high risk of transmission of viruses including hepatitis B virus (HBV) and hepatitis C virus (HCV) and human immunodeficiency virus (HIV). 9 The procedure for inactivating viruses in plasma preparations was only introduced in 1986, which did not protect patients from transmission of viruses without a lipid envelope (e.g., hepatitis A virus) or transmission of prions (Creutzfeldt–Jakob disease). 9 No case of transmission of infectious particles by latest generation recombinant FVIII/FIX concentrates has been described. 10

## Materials and Methods

This cross-sectional study was conducted with the cooperation of the Department of Conservative Dentistry and Endodontics and the Department of Haematology and Bone Marrow Transplantation. The study consisted of a questionnaire study and a clinical study. Prior to the study, the approval of the local Bioethics Committee (number 628/20 with amendments nr210/21) for the study protocol and survey was obtained. The study was conducted between September 2020 and June 2021. The study group included adult patients, aged between 18 and 70 years, under the care of the Department of Haematology and Bone Marrow Transplantation in whom congenital HA or HB was diagnosed on the basis of the clinical picture and currently available laboratory tests. The exclusion conditions were the presence of another acquired or congenital bleeding disorder, such as factor VII or other coagulation factor deficiency. A clinical examination of the oral cavity of patients with congenital hemophilia was performed in the outpatient clinic at the Department of Hematology and Bone Marrow Transplantation during a routine periodic health evaluation. The control group consisted of healthy volunteers, matched according to age and gender and examined in the Department of Conservative Dentistry and Endodontics. Seventy-seven patients with congenital hemophilia and 50 patients without congenital hemorrhagic diathesis were included in the study as the control group. The condition for including the patient in the study was age above 18 years. The patients were randomly selected. The conditions for excluding the patient from the control group were congenital or acquired hemorrhagic diathesis, age below 18 years, or lack of consent to participate in the study. The clinical dental examination of patients from both groups was performed by the same dentist. Informed written consent was obtained from all participants prior to the study.

### Characteristics of the Subjects in the Study Group

The study included 77 patients aged 18 to 70 years with known hemophilia. A total of 64 patients with HA and 13 patients with HB were studied. Almost 70% of the patients were patients with severe hemophilia with deficient coagulation factor activity below 1%, while patients with mild hemophilia (defined as a factor activity level >5 percent of normal) comprised almost 17% ( Table 1 ). Men constituted 100% of the study group. All patients were male with a median (Me) age of 35 years (range 18–70 years). There was no statistically significant difference found between the ages of patients with HA and HB and hemophilia severity (mild, moderate, and severe). Only one of the patients studied was edentulous. Of the study group, 44% of patients were treated “on demand” in the case of bleeding, whereas the rest of the study group received prophylactic therapy (factor administration, in the absence of bleeding, three times weekly 25 IU/kg FVIII concentrate in HA and twice weekly 30 IU/kg FIX in HB to prevent further morbidity). Patients had been treated with cryoprecipitate or fresh frozen plasma in childhood. None of the patients studied had been infected with HIV. Forty percent of the patients had anti-HCV antibodies, while anti-hepatitis B core total antibodies were detected in 42% of the patients. Anti-hepatitis B surface antibodies, indicating the acquisition of immunity to HBV by vaccination or history of disease, were detected in 81% of patients.

**Table 1:** Characteristics of the study group including the type and severity of hemophilia and the type of management

		*n*	%
Type of hemophilia	A	64	83.17
	B	13	16.88
Routine managment	On-demand therapy	34	44.16
	Secondary prophylactic therapy	43	55.84
Severity of hemophilia	Severe	53	68.83
	Moderate	11	14.29
	Mild	13	16.88

### Characteristics of the Control Group

The control group consisted of 50 healthy volunteers, men without any hereditary bleeding disorder or any other coagulation disorder problems. The Me age was 29.5 years, in the range of 21 to 75 years.

### Dental Clinical Examination

A clinical dental examination was performed on all patients, including dental caries incidence based on decayed, missing, and filled teeth (DMFT) and decayed, missing, and filled permanent teeth or surface (DMFS) indices, caries treatment (treatment index), simplified oral hygiene index status (OHI-s) according to Greene and Vermillion 11 and Approximal Plaque Index (API), and periodontal status (bleeding on probing [BoP]). The examinations were under artificial light, using disposable diagnostic instruments (mirror, probe) and a periodontal probe type WHO 621, ending in a ball with a diameter of 0.5 mm.

### Questionnaire

The questionnaire study was conducted on the basis of the author's survey card; it included questions concerning subjective assessment of oral health, pro-health habits related to prevention of oral diseases, and any occurring symptoms of hemophilia. In addition, socio-demographic data were taken into account.

### Statistical Analysis

Standard descriptive statistics were used to present baseline demographics, with categorical variables presented as frequencies ( *n* ), and proportions and numerical variables presented as Me and standard error (SE), and these were presented for normally distributed variables, otherwise, medians, and SE were used. The Shapiro–Wilk test was performed to assess normality. To compare differences between the groups, the chi-square test was used for categorical variables and the Mann–Whitney U test for continuous variables. The tests used in the study to compare more than two variables were the Kruskal–Wallis tests and the Dunn's test. A *p* -value below 0.05 was regarded as statistically significant. The statistical analyses were performed with STATISTICA 13.3 (Statsoft, Inc., Cracow, Poland).

## Results

**Fig. 1:**
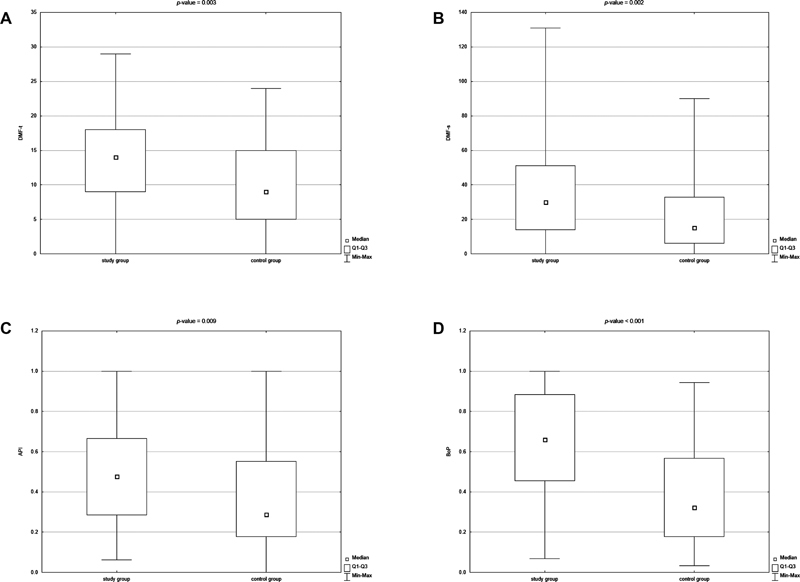
( **A** - **D** ) Box plots for decay–missing–filled teeth index (DMFT), decay–missing–filled surfaces index (DMFS), Approximal Plaque Index (AP), and bleeding on probing (BoP) in the test and control groups ( *p* -Value <0.05 according to the Mann–Whitney test).

The results of the study are presented in seven tables and seven figures. Table 2 shows the medians and upper and lower quarters of individual dental indices and their components in the study and control groups. There were statistically significant differences in the values of DMFT, DMFS, BoP, and the API index. Fig. 1 shows the graphical differences between the study group and the control group in the values of dental indicators related to caries incidence (DMFT and DMFS), oral hygiene status (API), and periodontal status (BoP). Table 3 refers to the verbal interpretation of the API index.

**Table 2:** Comparison of individual dental indicators and their components between the study group and the control groups (median; Q1–Q3)

	Study group ( *n* =77)	Control group ( *n* =50)	*p* -Value
	M [Q1–Q3]	M [Q1–Q3]	
D-t	1 [0–3]	1 [0–3]	0.268
M-t	1 [0–5]	0.5 [0–3]	0.184
F-t	7 [2–12]	5.5 [3–9]	0.478
DMFT	14 [9–18]	9 [5–15]	0.003 a
D-s	2 [0–5]	1 [0–5]	0.281
M-s	5 [0–25]	0 [0–15]	0.171
F-s	12 [3–20]	8 [5–14]	0.305
DMFS	30 [14–51]	15 [6–33]	0.002 a
Treatment index	0.86 [0.50–1.00]	0.89 [0.80–1.00]	0.168
BoP	0.66 [0.45–0.88]	0.32 [0.18–0.57]	<0.001 a
DI	0.17 [0.00–0.50]	0.00 [0.00–0.33]	0.124
CI	0.00 [0.00–0.17]	0.00 [0.00–0.17]	0.553
OHI-s	0.33 [0.00–0.67]	0.17 [0.00–0.33]	0.141
API	0.48 [0.29–0.67]	0.29 [0.18–0.55]	0.009 a

Abbreviations: API, Approximal Plaque Index; BoP, bleeding on probing; CI, Calculus index; D-t, decayed teeth; DI, debris index; DMFS, decayed, missing, filled surfaces; DMFT, decayed, missing, filled teeth; D-s, decayed surfaces; F-s, filled surfaces; F-t, filled teeth; M-s, missing surfaces; M-t, missing teeth; OHI-s, simplified oral hygiene index.

a *p* -Value < 0.05 for the Mann–Whitney test.

**Table 3:** Oral hygiene (based on API index) in patients with and without congenital hemophilia

	Optimal	Good	Sufficient	Insufficient	
Study group *n* = 74	18.92	24.32	36.49	20.27	*p* -Value 0.049 a
Control group *n* = 50	42.00	16.00	28.00	14.00	

Abbreviation: API, approximal plaque index.

a *p* -Value <0.05 for the chi-square test.

There were no statistically significant differences between the caries incidence and oral hygiene of patients according to the type of clotting factor deficiency (HA or HB) ( Table 4 ) and the type of prophylaxis used (prophylactic/“on-demand” agent) ( Table 5 ).

**Table 4:** Comparison of individual dental indicators and their components, between hemophilia A and hemophilia B (median; Q1–Q3)

	Hemophilia A ( *n* =64)	Hemophilia B ( *n* =13)	*p* -Value
	M [Q1–Q3]	M [Q1–Q3]	
D-t	1.5 [0–3.5]	0 [0–2]	0.081
M-t	0.5 [0–5]	2 [0–5]	0.434
F-t	7 [2–12]	9 [5–13]	0.217
DMFT	14 [8.5–17.5]	15 [9–18]	0.984
D-s ^e^	2.5 [0–6.5]	0 [0–5]	0.188
M-s	0 [0–22.5]	10 [1–25]	0.225
F-s	11 [3–20]	14 [7–25]	0.288
DMFS	25 [14–50]	37 [14–55]	0.812
Treatment index	0.84 [0.40–1.00]	1.00 [0.82–1.00]	0.071
BoP	0.67 [0.50–0.90]	0.62 [0.38–0.81]	0.271
DI	0.00 [0.00–0.50]	0.33 [0.00–0.50]	0.295
CI	0.00 [0.00–0.17]	0.00 [0.00–0.00]	0.619
OHI-s	0.17 [0.00–0.67]	0.50 [0.00–0.67]	0.373
API	0.43 [0.28–0.67]	0.53 [0.36–0.67]	0.473

Abbreviations: API, Approximal Plaque Index; BoP, bleeding on probing; CI, Calculus index; D-t, decayed teeth; DI, debris index; DMFS, decayed, missing, filled surfaces; DMFT, decayed, missing, filled teeth; D-s, decayed surfaces; F-s, filled surfaces; F-t, filled teeth; M-s, missing surfaces; M-t, missing teeth; OHI-s, simplified oral hygiene index.

Note: *p* -Value <0.05 for the Mann **–** Whitney test.

**Table 5:** Comparison of individual dental indicators and their components according to the management of hemophilia, prophylaxis, versus on-demand therapy

	Prophylactic therapy ( *n* =43)	On-demand therapy ( *n* =34)	*p* -Value
	M [Q1–Q3]	M [Q1–Q3]	
D-t	1 [0–4]	1 [0–3]	0.945
M-t	1 [0–5]	0.5 [0–5]	0.854
F-t	9 [3–13]	4.5 [2–10]	0.112
DMFT	14 [10–18]	10.5 [6–19]	0.274
D-s	2 [0–10]	2 [0–4]	0.812
M-s	5 [0–25]	3 [0–20]	0.884
F-s	16 [6–21]	7.5 [3–17]	0.034 a
DMFS	34 [18–55]	18.5 [10–49]	0.218
Treatment index	0.89 [0.50–1.00]	0.83 [0.58–1.00]	0.441
BoP	0.64 [0.50–0.84]	0.67 [0.45–0.91]	0.601
DI	0.17 [0.00–0.33]	0.00 [0.00–1.00]	0.657
CI	0.00 [0.00–0.17]	0.00 [0.00–0.17]	0.588
OHI-s	0.25 [0.00–0.50]	0.33 [0.00–1.00]	0.504
API	0.43 [0.29–0.63]	0.53 [0.29–0.68]	0.557

Abbreviations: API,Approximal Plaque Index; BoP, bleeding on probing; CI, Calculus index; D-t, decayed teeth; DI, debris index; DMFS, decayed, missing, filled surfaces; DMFT, decayed, missing, filled teeth; D-s, decayed surfaces; F-s, filled surfaces; F-t, filled teeth; M-s, missing surfaces; M-t, missing teeth; OHI-s, simplified oral hygiene index.

a *p* -Value <0.05 for the Mann–Whitney test.

Table 6 shows a comparison of indicators with their components of dental caries incidence (DMFT and DMFS), caries treatment (treatment index), oral hygiene status (OHI-s, API), and periodontal status (BoP) between patients with mild, moderate, and severe hemophilia.

**Table 6:** Comparison of individual dental indicators and their components according to severity of hemophilia—mild, moderate, and severe

	Mild ( *n* =13)	Moderate ( *n* =11)	Severe ( *n* =53)	*p* -Value
	M [Q1–Q3]	M [Q1–Q3]	M [Q1–Q3]	
D-t	1 [0–2]	2 [0–3]	1 [0–3]	0.849
M-t	0 [0–2]	1 [0–4]	1 [0–6]	0.741
F-t	6 [4–8]	4 [1–12]	8 [2–13]	0.441
DMFT	9 [7–11]	14 [5–22]	14 [9–18]	0.165
D-s	2 [0–3]	3 [0–4]	2 [0–7]	0.946
M-s	0 [0–10]	5 [0–20]	5 [0–25]	0.780
F-s	8 [6–11]	5 [0–20]	14 [3–21]	0.179
DMFS	14 [10–32]	30 [9–80]	34 [17–55]	0.165
Treatment index	0.88 [0.67–1.00]	0.80 [0.40–1.00]	0.86 [0.50–1.00]	0.761
BoP	0.68 [0.67–0.89]	0.45 [0.38–0.64]	0.66 [0.45–0.90]	0.058
DI	0.00 [0.00–0.00]	0.67 [0.00–1.33]	0.17 [0.00–0.50]	0.025 a
CI	0.00 [0.00–0.17]	0.00 [0.00–0.33]	0.00 [0.00–0.17]	0.715
OHI-s	0.00 [0.00–0.33]	0.83 [0.17–1.50]	0.33 [0.00–0.50]	0.042 a
API	0.32 [0.22–0.55]	0.43 [0.36–0.67]	0.50 [0.29–0.70]	0.429

Abbreviations: API,Approximal Plaque Index; BoP, bleeding on probing; CI, Calculus index; D-t, decayed teeth; DI, debris index; DMFS, decayed, missing, filled surfaces; DMFT, decayed, missing, filled teeth; D-s, decayed surfaces; F-s, filled surfaces; F-t, filled teeth; M-s, missing surfaces; M-t, missing teeth; OHI-s, simplified oral hygiene index.

a *p* -Value <0.05 for the Kruskal–Wallis test.

**Fig. 2:**
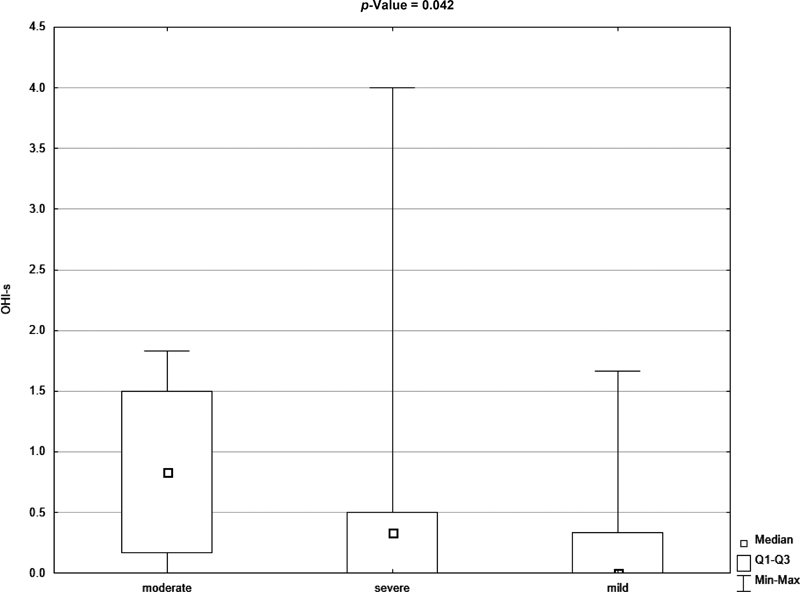
Box plot for oral hygiene index (OHI-S) in the severity of hemophilia (mild, moderate, and severe) ( *p* -value <0.05 according to the Mann–Whitney test).

**Fig. 3:**
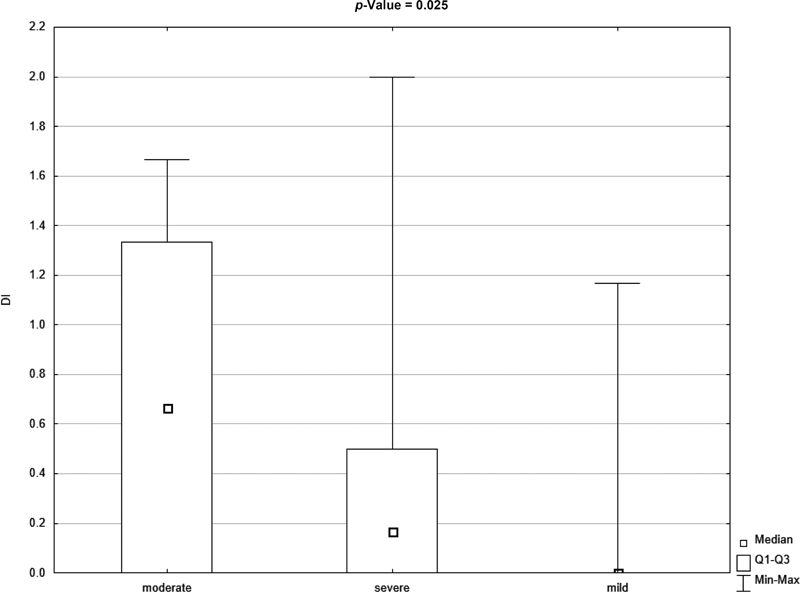
Box plot for debris index (DI) in the severity of hemophilia (mild, moderate, and severe) ( *p* -value <0.05 according to the Mann–Whitney test).

Figs. 2 and 3 show the graphical difference between debris index (DI) and OHI-S index values in patients with mild, moderate, and severe hemophilia.

Table 7 presents a comparison of indicators and their components of dental caries incidence (DMF and DMFS), caries treatment (treatment index), oral hygiene status (OHI-s, API), and periodontal status (BoP) depending on the age of the patient with hemophilia (18–24 years of age and over 34 years of age). The statistically significant differences were related to missing teeth, DMFT, missing surfaces, DMFS, OHI-s, and DI. There is a tendency to inferior hygiene in the interdental spaces (API) in patients with hemophilia over the age of 34 years.

**Table 7:** Comparison of individual dental indicators and their components according to severity of aged

	≤34 y old ( *n* =35)	>34 y old ( *n* =42)	*p* -Value
	M [Q1–Q3]	M [Q1–Q3]	
D-t	1 [0–3]	1.5 [0–3]	0.983
M-t	0 [0–0]	5 [1–10]	<0.001 a
F-t	8 [3–11]	6.5 [2–12]	0.967
DMFT	10 [6–14]	17 [13–22]	<0.001 a
D-s	2 [0–4]	2 [0–7]	0.837
M-s	0 [0–0]	20 [5–48]	<0.001 a
F-s	11 [5–17]	13.5 [3–26]	0.218
DMFS	17 [10–23]	49 [36–68]	<0.001 a
Treatment index	0.88 [0.40–1.00]	0.84 [0.52–1.00]	0.991
BoP	0.68 [0.58–0.91]	0.62 [0.41–0.77]	0.068
DI	0.00 [0.00–0.17]	0.33 [0.00–1.00]	<0.001 a
CI	0.00 [0.00–0.00]	0.00 [0.00–0.17]	0.283
OHI-s	0.00 [0.00–0.33]	0.50 [0.17–1.00]	<0.001 a
API	0.39 [0.23–0.63]	0.57 [0.36–0.86]	0.008 a

Abbreviations: API,Approximal Plaque Index; BoP, bleeding on probing; CI, Calculus index; D-t, decayed teeth; DI, debris index; DMFS, decayed, missing, filled surfaces; DMFT, decayed, missing, filled teeth; D-s, decayed surfaces; F-s, filled surfaces; F-t, filled teeth; M-s, missing surfaces; M-t, missing teeth; OHI-s, simplified oral hygiene index.

a *p* -Value <0.05 for the Mann–Whitney test.

Table 8 shows the results of the questionnaire study on the health-promoting habits related to the prevention of oral diseases. Statistically significant differences between the study and control group concerned the reason for the last visit to dental surgery, frequency of tooth brushing, use of mouthwash and flossing, preferred type of toothbrush for brushing, and professional dental plaque removal procedures.

**Table 8:** Comparison of oral hygiene habits in the study group and the control group

	Control group	Study group	*p* -Value
How often do you visit the dentist?			
Every 6 mo	30.0	48.0	0.168
Every year	44.0	27.3	
Every 2 y	8.0	6.5	
Less frequently	18.0	18.2	
What was the reason for the last visit?			
Consultation	8.0	19.5	0.001 a
Toothache/Gum problem	30.0	18.2	
Treatment continuation	20.0	29.9	
Dental check-up	42.0	22.1	
I don't remember	0.0	10.3	
Have you experienced oral pain in the past year?			
Yes	46.0	31.2	0.068
No	54.0	64.9	
I don't remember	0.0	3.9	
How do you rate your oral health?			
Good	60.0	39.0	0.066
Average	32.0	48.0	
Bad	8.0	13.0	
How often do you brush your teeth?			
Less frequently than once a day	8.0	3.9	0.040 a
Once a day	8.0	23.4	
Twice a day	72.0	58.4	
Three times a day	6.0	13.0	
After every meal	6.0	1.3	
How long do you brush your teeth?			
Half a minute	2.0	7.8	0.612
1 min	16.0	18.2	
2 min	40.0	37.7	
3 min or more	28.0	22.1	
I don't know	14.0	14.2	
Do you clean your tongue?			
Yes	48.0	45.4	0.779
No	52.0	54.6	
Do you use mouthwash?			
Yes	58.0	37.7	0.024 a
No	42.0	62.3	
Do you floss?			
Yes	46.0	15.6	<0.001 a
No	54.0	84.4	
What kind of tooth brush do you use?			
Manual	58.0	67.5	0.018 a
Sonic	8.0	18.2	
Electric	34.0	14.3	
Have you had professional teeth cleaning?			
Yes	80.0	31.2	<0.001 a
No	20.0	68.8	
Do your gums bleed during brushing?			
Yes	28.0	36.4	0.421
No	52.0	40.3	
In the past	20.0	23.4	

a *p* -Value <0.05 for the Chi-square test.

There was no correlation between the value of the BoP and socio-demographic factors (education/residence/age).

## Discussion

Congenital hemophilia is a rare, genetic disorder. Due to its rarity and lack of characteristic oral lesions, there are few studies evaluating the oral health of adult patients with congenital bleeding diathesis, especially hemophilia. Most studies to date, evaluating the oral health of patients with hemophilia, have been conducted on children. 12 13 14 15 16 17 18 19 20 21 22 23 The aim of the present study was to compare the pro-health habits, oral hygiene status, and dental status between individual types (A and B) and severity (mild, moderate, and severe) of hemophilia and to compare the obtained results with a healthy control group, matched according to age and gender.

The results of the study indicate a higher incidence of dental caries in patients with hemophilia compared with healthy patients. There were no statistically significant differences in caries intensity depending on the deficient clotting factor (HA and HB) and severity (mild, moderate or severe) of hemophilia, so it can be assumed that patients with hemophilia are more likely to develop carious foci than those without hemorrhagic diathesis, which is confirmed by a study of adolescents from Pakistan (mean age 16 years). 24 In Pakistan, the caries severity was assessed on the basis of the DMFT index, which was 2.07 in patients with severe hemophilia (deficient clotting factor level <2% of normal), compared with 0.95 for patients without congenital hemorrhagic diathesis. In this study, as in our study, the primary focus was on patients with severe hemophilia. The study by Azhar et al included patients with severe hemophilia only, in our study nearly 70% of patients had severe hemophilia.

Both the results of the present study and the study from Pakistan do not match the results obtained in the state of Karnataka (India), where 100 patients with HA and HB were studied—that study was conducted on adult and child patients (age range, 2–73 years). 12 The study group consisted of 59 adult patients and 41 children, in contrast to our study, where the inclusion condition was age over 18 years. Despite the fact that the researchers from India found worse oral hygiene statuses of patients with hemophilia compared with the group without congenital hemorrhagic diathesis (OHI-s of 2.176 and 1.721, respectively, with *p* = 0.03), there were no statistically significant differences in the values of the DMFT indices (3.51 for the group of patients with hemophilia and 3.50 for patients without congenital hemorrhagic diathesis [ *p* = 0.983]). The results contrasted with our results because, according to our study, the hygiene in the interdental spaces was worse in the study group (API 48% compared with 29% in the study group), but the difference in OHI-s values was not statistically significant. Interestingly, the study showed a higher level of anxiety before dental treatment for patients with hemophilia compared with the control group ( *p* = 0.003).

A study conducted in Vilnius (Lithuania), similarly to the study from India and as opposed to our research, negates the higher incidence of caries in patients with congenital hemophilia. 25 The study group included 49 adults and 27 children, and the age range of the study group was from 4 to 60 years. The DMFT for subjects with coagulation factor deficiency was 9.4, and for healthy subjects 9.3 ( *p* = 0.947). The lack of differences in the dental status of adult patients with and without hemophilia is also reported in a study conducted in Meshheda (Iran) on a population of adult and child patients with hemophilia (age range, 4–60 years and 7–34 years). 26 It is, however, essential that DMFT increased significantly with the patient's age in this study as in our study, DMFT and DMFS increased with age. Both, Lithuanian and Iranian researchers, unlike us, included patients under 18 years of age in the control group.

In Hannover (Germany), there were no statistically significant differences in caries incidence when the dental statuses of patients with hemophilia and other congenital hemorrhagic diatheses were compared with a population of patients without coagulation disorders. 27 The DMFT for patients with congenital hemorrhagic diathesis was 18, while for the patients in the control study group it was 15 ( *p* = 0.41). It is worth noting that the study group included only eight patients with HA and seven patients with von Willebrand disease. The Hannover researchers assessed patients with congenital bleeding disorders, and they did not focus as much as we did on a specific disease entity. In the opinion of the authors of this publication, the German study was based on a small number of patients and it can be assumed that when analyzing the dental status of a larger group of patients, different results are likely to be obtained. The authors are also of the opinion that the discrepancy in the results obtained between the study conducted in Poznan and the studies by Zaliuniene et al 25 and Kumar et al 12 may be due, among other things, to the very wide age ranges of the patients in the studies conducted in Lithuania and India. The DMFS/ DMFT ratios are calculated separately for permanent and deciduous teeth, which will result in the need for separate statistical analyses and a smaller study group. Furthermore, the first permanent teeth appear around 6and 7 years of age, which means that in some of the patients studied there may not have been time for caries to develop. 28

The authors of this publication would like to emphasize that they did not find other studies conducted exclusively on adult patients with congenital hemophilia, and their own study was performed on a large group of patients (77 patients) consisting of almost 70% of patients with a severe form of hemophilia. This has a positive impact on the value of the publication and indicates the need for further research in this direction. In all the studies cited above, the oral health status was analyzed in mixed groups—children and adults or hemophilia and von Willebrand disease, which resulted in the inclusion of a smaller number of adult patients with congenital hemophilia. Another strength of the study is the fact that not only young people but also patients over 60 years of age were analyzed. It is important that the center where the research was conducted is the only center in the Greater Poland voivodeship that implements the “National Program of Treatment of Patients with Hemophilia and Related Hemorrhagic Diathesis” for adult patients (I degree of referentiality). Despite the coronavirus disease 2019 pandemic, approximately 70% of patients with HA or HB, treated in the selected center, were successfully tested. The analyzed patients had different sociodemographic statuses, which significantly influences the patients' pro-health awareness and, thus, makes the study objective. Furthermore, a major advantage of this study is that it is a prospective analysis and included patients from one region, treated differently, and this was included in the final analysis. It is less clear how the choice of center affects the results obtained, and whether similar results would be obtained in patients under 18 years of age. Other limitations include the rarity of congenital hemorrhagic diathesis, the inability to conduct a clinical examination in dental surgery, and the inability to verify caries on radiographs.

The results of our study indicate the worse effectiveness of hygienic procedures performed in interdental spaces by patients with congenital hemophilia compared with healthy patients. Moreover, according to the survey, patients with congenital hemophilia use professional hygienic procedures less frequently than patients without bleeding diathesis. Due to the possibility of plaque accumulation in non-stitched contact spaces, according to the authors, both the DMFT index and the DMFS index are important for studies comparing dental status, as only the latter takes into account the number of tooth surfaces affected by caries. The inferior effectiveness of hygienic procedures and the difference in pro-health habits may be a direct cause of the poorer dental status of patients with congenital hemophilia. This can be confirmed by the statistically significant difference between the patient's age and the value of OHI-s, DI, DMFT, DMFS indexes. There is a tendency to inferior hygiene in the interdental spaces (API) in patients with hemophilia over the age of 34 years. However, it should be emphasized that the goal of the center where the research was conducted is to provide multidisciplinary medical care for patients with congenital bleeding disorders so that, in accordance with the WFH 2020 directives, the health statuses of people with hemophilia do not differ from the general population. However, the center has only been running since May 2019, and the improvement of health is a long-term process that requires not only appropriate treatment but also changes in patients' pro-health habits. In the authors' opinion, appropriate health education and a focus on dental prophylaxis for patients with congenital hemophilia will affect oral health status—thus, the incidence of caries may depend on the region in which the research is conducted. Due to the fact that Greater Poland is one of 16 provinces in Poland and accounts for approximately 10% of the country's area, the oral health of patients with hemophilia in other parts of the country may differ from the results obtained, which may be both a disadvantage and an advantage of the study. A limitation of the study is the wide age range of patients and the inability to divide patients into equal age groups, which is dictated by the rare occurrence of hemophilia, and thus the inability to collect an ideal study group. This study began to reveal differences that may exist in the oral health of patients with congenital hemophilia compared with patients without a congenital bleeding disorder. Unanswered questions also began to appear in the study. To what extent is the incidence of caries related to hemophilia and to what extent is it due to poorer oral hygiene, and will the duration of the disease affect the health of the oral cavity? According to the authors, further studies are needed to assess the health of the oral cavity, caries incidence, and health-promoting habits of patients with congenital hemophilia of different ages and origins.

The difference in the health-promoting habits of patients with congenital hemophilia compared with those without hemorrhagic diathesis may be directly related to bleeding occurring in the oral cavity. Statistically significant differences ( *p* < 0.001) were found in the values of the BoP between patients in the study group and the control group. According to the authors, the reluctance of patients with hemophilia to use flossing, scaling, and sandblasting may be due to the fear of excessive bleeding. Reductions in the DMFT and DMFS indices can be achieved only through prophylactic measures; therefore, patient education and the shaping of behaviors affecting the amount of cryogenic bacteria in the oral cavity seem to be particularly important in the group of patients with congenital hemophilia. Centers treating younger patients will play an important role. It should be remembered that a condition of success is the encouragement of good health-promoting habits at the earliest stage of life. A priority seems to be the education of parents of children with congenital hemophilia, as the level of knowledge of the caregivers will have an impact on the health of the child's oral cavity. 29 30 Centers treating adult patients should continue health education and, in addition, one of the goals should be to educate patients about factors that may affect the development of caries, such as smoking 31 32 or diet. 33 34 Patients with congenital hemophilia should be recommended follow-up visits to the dentist at least every 6 months, as well as regular hygienization in the dental surgery. Contact with a professional dental hygienist at the earliest possible stage of life seems imperative to develop the appropriate hygiene habits related to the oral cavity. It is recommended to individually select the optimal technique of brushing teeth and brushing teeth at least twice a day, using dental floss and mouth rinses.

A higher BoP value may also be an indirect factor for the differences in the reasons for making dental appointments in patients with VIII/IX coagulation factor deficiency compared with patients without hemorrhagic diathesis—difficulties arising during the dental treatment of patients with coagulation disorders will often necessitate multi-visit treatment. For example, the occurrence of excessive bleeding during the treatment of a Class II Black's cavity may prevent the treatment area from being kept dry and, therefore, the use of a final filling. In addition, some dental procedures will be postponed by the dentist because of the need to supplement a deficient clotting factor, for example, before a tooth extraction or before anesthesia of the inferior alveolar nerve. 35 It seems clear that differences in the reason for appointments will also be related to different incidences of caries.

## Conclusions

Patients with congenital hemophilia may present with the same oral diseases as patients without congenital hemorrhagic diathesis. Due to the possibility of gingival bleeding, patients will be apprehensive about certain prophylactic treatments. Health-promoting habits have a direct impact on the development of the oral microflora and, thus, on dental and periodontal health. In view of the above, the basis for dental treatment of patients with coagulation disorders should be prophylaxis and appropriate health education introduced at the earliest possible stages of life.
